# Emotional Suppression and Psychological Well-Being in Marriage: The Role of Regulatory Focus and Spousal Behavior

**DOI:** 10.3390/ijerph19020973

**Published:** 2022-01-15

**Authors:** Unji An, Haeyoung Gideon Park, Da Eun Han, Young-Hoon Kim

**Affiliations:** 1 Department of Psychology, Yonsei University, Seoul 120-749, Korea; unji0604@hanmail.net; 2 Department of Psychology, University of Toronto, Mississauga, ON L5L 1C6, Canada; gideonpa@gmail.com; 3 Department of Psychology, University of Illinois at Urbana-Champaign, Champaign, IL 61820, USA; daeuneh2@illinois.edu

**Keywords:** emotional suppression, regulatory focus, spousal behavior, psychological well-being, marital satisfaction

## Abstract

Emotional suppression has been considered a critical factor in determining one’s mental health and psychological well-being in intimate relationships such as marriage. The present study aimed to delineate the nuanced association between emotional suppression and psychological well-being in marriage by considering two critical factors: (a) individual differences in motivational orientation and (b) the perceived level of a partner’s emotional suppression. A set of two online survey studies were conducted on a large sample of married participants. The participants were asked to indicate (a) their own level of emotional suppression, (b) the perceived level of their spouse’s emotional suppression, (c) relationship motivation, and (d) satisfaction with marital life. The results consistently indicated that for prevention-focused individuals being emotionally suppressive was associated with greater marital satisfaction, but only for those who perceived their spouses as also emotionally suppressive. Conversely, for promotion-focused individuals, being less emotionally suppressive was associated with greater marital satisfaction, but again, only for those who perceived their spouses as also being less emotionally suppressive. These findings provide insights into research on emotion regulation and self-regulatory strategies in influencing psychological well-being and mental health in an intimate relationship.

## 1. Introduction

In marriage, some spouses tend to overtly express their feelings to actively resolve issues and promote shared feelings of intimacy [1,2]. On the other hand, some are inclined to inhibit their emotional expressions to avoid disruptions and maintain a stable relationship [3]. However, which emotional strategy would be more helpful for a successful marriage: overtly displaying one’s emotions or being covert about one’s emotions?

Emotional suppression, defined as the conscious inhibition of expressive behaviors during emotional arousal [4], has been considered to have a noteworthy effect on an individual’s mental health and psychological well-being in close relationships [5,6]. The past literature has mostly discussed its potential adverse effects on intimate relationships such as marriage. For instance, researchers found that habitual suppression of emotional expression was associated with lower relationship satisfaction among both romantic and married couples [7,8,9]. Hence, in this vein, it would seem that minimal concealment in the expression of one’s emotions should help promote marital success.

Meanwhile, another stream of research findings suggests that emotional suppression may entail some adaptive social functions with positive implications. For instance, longitudinal research on marriage has revealed that the quicker the spouses’ emotional suppression, the greater the marital satisfaction of both partners [10]. Furthermore, scholars have found that people who intend to foster stable relationships value emotional suppression more in order to achieve interpersonal harmony; thus, emotional suppression is associated with greater well-being [11,12]. In line with these findings, one of the most recent studies on emotional regulation found the absence of negative consequences for emotional suppression within marriage [13].

Based on this analysis of the literature, it remains unclear why emotional suppression appears detrimental in some cases but helpful in others. To delineate the nuanced associations between emotional suppression and psychological well-being in marriage, the present study emphasizes the importance of considering two critical factors: (a) individual differences in motivational orientation in terms of prevention and promotion motivations and (b) the perceived level of suppression of the corresponding partner.

### 1.1. Emotional Suppression and Regulatory Focus

To resolve the inconsistency among the effects of emotional suppression on satisfaction in marriage, the present study suggests the need to consider which emotional behaviors are more adaptive for people with different motivational goals in their relationships [14]. Since individuals set distinctive motivational goals in marriage, such as preventing conflicts or promoting intimacy [15,16], the effects of emotional suppression on one’s relationship satisfaction may differ depending on the extent to which their emotional behavior aligns with their ultimate goals. In this regard, the present study discusses the long-standing theoretical framework of motivational systems: regulatory focus theory [17].

According to regulatory focus theory [17], individuals have two distinct motivational systems: prevention and promotion. Prevention-focused individuals are mainly motivated by their desire to avoid potential costs, whereas promotion-focused individuals are generally driven by their desire to achieve potential rewards. Based on these differences, individuals adopt contrasting strategies to achieve their divergent goals [18].

Specifically, prevention-focused individuals aim to escape from negative outcomes and, thus, primarily adopt avoidance strategies [17,18,19]. Since prevention-motivated individuals prefer avoiding negative experiences (i.e., conflicts and disruptions) over obtaining positive experiences (i.e., intimacy and affection) [15,20], they place more value on maintaining relationship stability than enhancing their relationship when determining satisfaction [21]. In such situations, emotional suppression is considered a positive avoidance strategy for those with prevention motivation, as the inhibition of emotional behaviors is assumed to be the safest way to maintain the status quo and ensure that nothing goes wrong within the relationship [14,22]. Thus, it could be predicted that emotional suppression would lead to greater marital satisfaction for prevention-focused individuals.

In contrast, promotion-focused individuals seek positive outcomes and, thus, mainly adopt approach strategies [17,18,19,20]. They consider it more important to attain potential benefits, such as feeling uplifted and affectionate, when pursuing relationship satisfaction [15]. In such cases, overtly conveying one’s emotions constitutes an effective way of working toward attaining better outcomes, such as resolving conflicts and increased feelings of closeness, rendering emotional suppression counterproductive [23]. Hence, as opposed to prevention-focused individuals, being less emotionally suppressive would lead to greater marital satisfaction, as concealing emotions does not align well with their relationship goals.

### 1.2. Regulatory Fit: Congruence between Partners

In line with the suggestion that one’s regulatory focus plays a significant role in predicting the adaptiveness of their emotional strategies, the current study also suggests the value of considering another critical factor within the interactive dynamics of marital relationships: perceptions of the partner’s behavior [24]. Emotional suppression occurs not in a vacuum but mostly within the context of social interactions [24,25,26], where the perceived behavior of the partner should also be crucial when determining whether one’s own emotional strategy should be adaptive. Although studies have shown that a spouse’s level of emotional suppression could significantly affect one’s satisfaction in marriage [8,11], scholars have yet to directly examine the interactive effects between one’s own and one’s perceived level of their spouse’s emotional suppression. Therefore, the present study investigates how one’s perceptions of their partner’s emotional suppression also shape the adaptiveness of their own within marriage.

Specifically, we predict that an individual’s adaptive emotional behavior would most likely lead to greater marital satisfaction when their spouse’s behavior is also perceived as congruent (vs. incongruent) with their emotional strategy [27]. To support this prediction, research has emphasized the importance of the match between individuals’ motivational goals and their surrounding environment, which encourages their use of specific means to achieve such goals (i.e., regulatory fit [28]). Since individuals set distinctive goals (stability vs. advancement) in marriage and consistently monitor their spouse’s fulfillment of these motivational goals [29], the perception of a spouse’s pursuit of a joint goal should positively contribute to one’s marital happiness [27]. Hence, for prevention-motivated individuals, emotional suppression would be associated with higher marital satisfaction when their respective spouses are also perceived as being emotionally suppressive. Similarly, for promotion-motivated individuals, being more overt with one’s emotions would be associated with more positive outcomes when their respective spouses are also perceived as less emotionally suppressive.

In contrast, we predict that the positive effects of one’s emotional behavior may be attenuated when their spouse’s behavior is perceived as incongruent with their own, as their spouse’s opposite behavior may offset their effort to either prevent risks or achieve rewards within the relationship [27]. For prevention-focused individuals, despite the effectiveness of emotional suppression for avoiding risks and maintaining a stable dynamic [30], the positive impact of such efforts may be negated when their spouse is perceived as being overtly expressive with their emotions, which disrupts the desired stability. Similarly, promotion-focused individuals’ efforts to actively communicate their emotions and achieve better outcomes may be mitigated when their spouse is perceived as withholding their emotions to prevent their relationship from improving. Therefore, we predict that the positive impact of adaptive behaviors for both prevention- and promotion-focused individuals would be diminished when they perceive their spouse as behaving in the opposite direction from their marital goals.

### 1.3. Overview of the Present Study

This current research uses a set of two studies that consider one’s regulatory focus and the perceived level of the marital partner’s emotional suppression to outline the differential effects of emotional suppression within marriage. We hypothesize that for prevention-focused individuals, being emotionally suppressive would be associated with greater marital satisfaction, with stronger effects when they perceive their spouses as the same. In a similar vein, for promotion-motivated individuals, we predict that less emotional suppression would be associated with greater marital satisfaction, with stronger effects when they perceive their spouses as less emotionally suppressive as well. We predict that the positive impacts of the regulatory strategies employed by both prevention- and promotion-focused individuals would be offset and negated when their spouses’ behaviors are perceived as incongruent with their regulatory strategy.

One must note that our hypotheses were tested using individuals’ reports on their own and partners’ levels of emotional suppression rather than utilizing dyadic reports from both spouses. Several reasons support this decision. First, past research has found that individuals’ reports of their partners’ emotional suppression levels did not significantly correlate with their partners’ self-reports of their emotional suppression levels [24]. This confers that couples might not be able to accurately gauge or agree on each other’s emotional suppression levels due to the introspective and covert nature of emotional suppression. Second, it has been shown that individuals’ perception of their partners’ emotional suppression is a stronger predictor of their relationship satisfaction than their partners’ self-reported levels of emotional suppression [24]. Hence, in testing our prediction on the role of a partner’s level of emotional suppression in regulatory fit, individual’s perception of their partner’s emotional suppression level was considered both appropriate and sufficient for the present research.

## 2. Study 1: Methods and Materials

### 2.1. Participants and Procedures

The present study tested its hypotheses as part of a larger research project on the psychological well-being of married individuals from South Korea. Data were collected from an online dataSpring survey administered to a total of 1179 married participants (633 females, 546 males; mean age = 42.09 years; *SD* = 7.45 years). A post hoc power analysis via G*Power [31] revealed that such a sample size allows for the detection of small effect sizes with 95% statistical power at a 0.05 alpha level. After providing informed consent, the participants completed several self-report measures presented through a secure website. All procedures were approved by the university’s institutional review board for ethics.

### 2.2. Measures

#### 2.2.1. Emotional Suppression toward One’s Spouse

Participants answered the suppression subscale of the Emotion Regulation Questionnaire (ERQ) [32] to describe their habitual level of emotional suppression within marriage. The subscale consists of four items rated on a seven-point Likert scale from 1 (strongly disagree) to 7 (strongly agree). Each item was revised to specifically assess emotional suppression in the context of marital relationships (e.g., “I control my emotions by not expressing them to my spouse”). The items in the present sample demonstrated adequate reliability (Cronbach’s alpha = 0.84).

#### 2.2.2. Perceived Level of a Spouse’s Emotional Suppression

The perceived level of a spouse’s emotional suppression was evaluated using the same suppression subscale [32]. The same four items rated on a seven-point Likert scale were then revised to determine the spouses’ suppression levels instead of the participants’ (e.g., “My spouse controls his/her emotions by not expressing them to me”). In the present sample, Cronbach’s alpha coefficient for this scale was 0.84.

#### 2.2.3. Regulatory Focus in Marital Relationships

Although individuals may have varying degrees of prevention and promotion motivations, the relative extent to which one leans toward one motivation in relation to the other ultimately determines an individual’s behaviors [19]. Therefore, a set of two face-valid items was used to directly assess the extent to which the participants endorsed prevention motivation relative to promotion motivation within marital relationships. This scale also adopted a seven-point Likert rating system from 1 (strongly disagree) to 7 (strongly agree) for the following statements: “I am more oriented toward preventing negative outcomes in my relationship than I am toward achieving positive outcomes” and “I am striving to protect my relationship more than I am attempting to enhance my relationship” (*r* = 0.84, *p* < 0.001). Higher scores indicate participants’ higher motivation via prevention than promotion.

#### 2.2.4. Marital Satisfaction

The Quality Marriage Index [33], which contains six items rated on a seven-point Likert scale from 1 (strongly disagree) to 7 (strongly agree), was used to measure the participants’ marital satisfaction (e.g., “My relationship with my partner makes me happy”). The items indicated adequate reliability and validity during the initial development by Norton [33]. Following the methods from previous research [34], all six items were averaged to create a single index, in which higher scores indicate greater marital satisfaction. This scale displayed a high level of reliability in our sample (Cronbach’s α = 0.96). All items in the present study were translated and back-translated into Korean.

## 3. Study 1: Results

Table 1 shows the means and standard deviations for the variables in Study 1 according to the age groups of the participants, and Table 2 shows the correlation coefficients between the main variables. To test our hypothesis, we conducted hierarchical regression analysis following guidance from Baron and Kenny (1986) [35]. The first step independently contained the mean-centered emotional suppression of the self, perceived level of a spouse’s emotional suppression, and regulatory focus. The second step added possible two-way interaction terms between the variables. Finally, the third step incorporated a three-way interaction between the variables (participant’s emotional suppression × perceived level of a spouse’s emotional suppression × regulatory focus).

**Figure 1:**
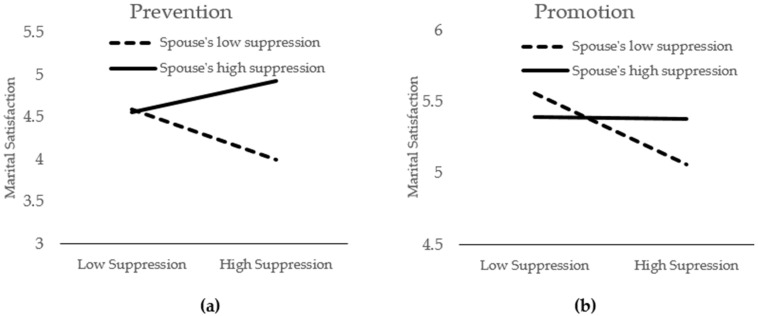
Three-way interaction effects of one’s own level of emotional suppression × perceived level of a spouse’s emotional suppression × regulatory focus on one’s marital satisfaction. (**a**) Two-way interaction effects of one’s own level of emotional suppression × perceived level of a spouse’s emotional suppression for prevention-focused individuals. (**b**) Two-way interaction effects of one’s own level of emotional suppression × perceived level of a spouse’s emotional suppression for promotion-focused individuals.

Table 3 displays the full regression values. Regarding marital satisfaction, the main effects of the three variables explained 13% (*p* < 0.001) of the variance in Step 1, whereas the three sets of two-way interactions contributed an additional 7% (*p* < 0.001) of variance in Step 2. Finally, the three-way interaction explained an additional 1% (*p* < 0.01) of variance. In Step 3, the results indicated the significant main effects of emotional suppression of the self (*b* = −0.07, *p* = 0.02), emotional suppression of the spouse (*b* = 0.10, *p* < 0.001), and regulatory focus (*b* = −0.27, *p* < 0.001). In addition, the interaction effects of emotional suppression of the self × emotional suppression of the spouse and emotional suppression of the self × regulatory focus were also significant (*b* = 0.12, *p* < 0.001, and *b* = 0.05, *p* < 0.001, respectively). However, these main effects and two-way interactions are qualified by the presence of significant three-way interactions (*b* = 0.03, *t*(1171) = 2.88, *p* < 0.01, 95% CI = [0.01, 0.04]), as seen in Figure 1. When gender was included in the model for a four-way interaction, no significant terms emerged, indicating that the interaction effects did not significantly differ by gender (*b* = 0.02, *p* = 0.40). Further, the three-way interaction also remained significant when marital duration was included in the regression model (*b* = 0.03, *t*(1170) = 2.83, *p* < 0.01, 95% CI = [0.01, 0.04]).

Decomposing the three-way interaction, we first examined the participants who were more motivated by prevention than promotion (evaluated at +1 *SD* from the mean; hereafter, prevention-motivated individuals). For prevention-motivated individuals, the interaction between one’s own level of emotional suppression and the perceived level of their spouse’s emotional suppression was statistically significant for marital satisfaction (*b* = 0.16, *t*(1171) = 7.14, *p* < 0.001). We then interpreted these significant interaction terms by plotting the scores for marital satisfaction at two data points, namely, one standard deviation above (+1 *SD*) and one standard deviation below (−1 *SD*) the means of the perceived spousal emotional suppression. As predicted, for prevention-focused individuals who perceived their spouses as highly emotionally suppressive, the suppression of one’s emotions was associated with higher marital satisfaction (*b* = 0.15, *t*(1171) = 3.25, *p* < 0.01, 95% CI = [0.06, 0.24]). However, for those who viewed their spouses as less emotionally suppressive, their own levels of suppression were associated with lower marital satisfaction (*b* = −0.24, *t*(1171) = −5.19, *p* < 0.001, 95% CI = [−0.33, −0.15]).

For the participants who were more motivated by promotion than prevention (evaluated at −1 *SD* from the mean; indicated from this point as promotion-motivated individuals), the interaction between one’s own level of emotional suppression and the perceived level of their spouse’s emotional suppression was also statistically significant regarding marital satisfaction but with significantly different patterns (*b* = 0.08, *t*(1171) = 3.03, *p* < 0.01). Again, significant interaction terms were interpreted by plotting the predicted values for satisfaction and calculating simple slopes at two data points, namely, high (+1 *SD*) and low (−1 *SD*) levels of spousal emotional suppression. Consistent with our prediction, for promotion-focused individuals who viewed their spouses as less emotionally suppressive, being less emotionally suppressive themselves was associated with higher marital satisfaction (*b* = −0.20, *t*(1171) = −4.57, *p* < 0.001, 95% CI = [−0.28, −0.11]). However, for those who perceived their spouses as highly emotionally suppressive, being less suppressive of emotions themselves was no longer associated with greater marital satisfaction (*b* = −0.01, *t*(1171) = −0.11, *p* = 0.91, 95% CI = [−0.12, 0.11]).

## 4. Study 2: Overview

Given that the results of Study 1 mostly supported our predictions, we conducted Study 2 to enhance the generalizability and robustness of our findings. First, since Study 1 was limited to a South Korean sample, in Study 2, we added a large sample from the United States to determine whether the results could be generalized to a broader cultural sample. Second, given that our regulatory focus measure in Study 1 was newly developed with only two items, we used a more highly validated and commonly used scale in Study 2.

Using a between-subject design, Study 2 also sought to account for the effects of emotional valence by including conditions that separately pertain to the suppression of positive and negative emotions. While the original emotional suppression scale [32] does not differentiate between suppressions of the different valence of emotions, this might be important to consider for the present research, as each regulatory focus is associated with a different valence of outcomes (absence of negative outcomes vs. presence of positive outcomes). Despite not clearly establishing any prior predictions, our exploratory aim was to consider any potential differences that might appear between the suppression of positive and negative emotions.

## 5. Study 2: Methods and Materials

### 5.1. Participants and Procedures

A priori sample size calculation via G*Power [31] revealed a sample requirement of at least 103 participants for each condition to detect small effect sizes of r = 0.15 with 80% statistical power at the 0.05 alpha level. Accordingly, we recruited a total of 968 married Americans (203 males, 250 females; mean age = 41.00 years; *SD* = 11.11 years) and Koreans (199 males, 316 females; mean age = 42.62 years; *SD* = 8.41 years) through the online survey services of Amazon’s Mechanical Turk and dataSpring, respectively. This sample size allowed six conditions (Country; America, Korea × Valence; general, positive, and negative) to include between 136 and 179 participants, providing sufficient power for the study. After providing informed consent, the participants completed several self-report measures on a secure website. In the online questionnaire, two attention check items (e.g., please indicate “strongly agree” for this question) were included, and the time spent completing the survey was recorded. For validity, the final data excluded the samples that (1) failed attention checks, (2) did not spend the appropriate amount of time to complete the survey, (3) completed the survey more than twice, or (4) withdrew before completing the survey. All procedures were approved by the university’s institutional review board for ethics.

### 5.2. Measures

#### 5.2.1. Emotional Suppression toward One’s Spouse

The suppression subscale of the ERQ [32] in Study 1 was applied with additional modifications. First, to specifically evaluate the suppression of emotions that emerge within the context of marriage, the term “emotions” in each item was revised to “emotions that I have experienced in my relationship with my spouse”. Second, to explore the potential differences between the effects of positive and negative emotional suppression, the participants were randomly assigned to one of three conditions, which varied according to the valence of emotions to which the suppression items referred (general emotion, positive emotion, and negative emotion). The first condition (i.e., general emotional suppression condition) measured one’s suppression of general emotions using the same four-item format as the ERQ’s original suppression subscale [32]. One example item is “I control the emotions that I have experienced in my relationship with my spouse by not expressing them to my spouse” (Cronbach’s α = 0.86 for both Koreans and Americans). The second condition (i.e., positive emotional suppression condition) used four items referring to the suppression of only positive emotions (e.g., “I control the positive emotions that I have experienced in my relationship with my spouse by not expressing them to my spouse”; Cronbach’s α = 0.96 and 0.93 for Koreans and Americans, respectively). The third condition (i.e., negative emotional suppression condition) has four items pertaining to the suppression of only negative emotions (e.g., “I control my negative emotions that I have experienced in my relationship with my spouse by not expressing them to my spouse”; Cronbach’s α = 0.96 and 0.98 for Koreans and Americans, respectively). All items were rated on a seven-point Likert scale from 1 (strongly disagree) to 7 (strongly agree).

#### 5.2.2. Perceived Level of a Spouse’s Emotional Suppression

The perceived level of a spouse’s emotional suppression was assessed with identical modifications and procedures as above. Items for measuring the participants’ own levels of emotional suppression and the perceived level of spousal emotional suppression were presented in the same format, in which, for instance, the participants assigned to the positive emotional suppression condition also reported the level of positive emotional suppression of their spouses. Cronbach’s alpha coefficients in the present sample ranged between 0.84 and 0.96, showing sufficient estimation for the analysis.

#### 5.2.3. Regulatory Focus in Marital Relationships

The extent to which the participants prioritized prevention over promotion within the marital relationship was assessed using the Regulatory Focus in Relationship Scale [15]. The scale was chosen to specifically assess individuals’ motivations within the specific context of intimate relationships, as their motivational tendencies may differ according to context. This scale’s psychometric properties have been validated by several studies [15,36]. The measure consists of 15 items rated on a seven-point Likert scale from 1 (strongly disagree) to 7 (strongly agree): seven items measured prevention concerns in marriage, such as “In general, I am striving to protect and stabilize my relationships” (Cronbach’s α = 0.75 and 0.87 for Koreans and Americans, respectively), and eight items measured promotion concerns in marriage, such as “In general, I am striving to nurture, grow, and enhance my relationships” (Cronbach’s α = 0.90 and 0.91 for Koreans and Americans, respectively).

As described in Study 1, Study 2 also aimed to capture the relative extent to which one leans towards prevention motivation in comparison to promotion motivation [19]. Hence, following the methods from previous studies [19,37], an index of difference scores was computed by subtracting promotion motivation scores from prevention motivation scores. Higher scores on this measure reflect a relatively stronger focus on prevention than promotion. This index showed significantly high correlations with both prevention (r = 0.80, *p* < 0.001) and promotion (r = −0.70, *p* < 0.001) scales, respectively.

#### 5.2.4. Marital Satisfaction

As in Study 1, the Quality Marriage Index [33] was used to measure the participants’ marital satisfaction (Cronbach’s α = 0.97 for both Koreans and Americans). All items in the present study were translated into Korean using a back-translation procedure for Korean participants.

## 6. Study 2: Results

Table 4 presents the means and standard deviations for the variables in Study 2 according to the age groups of the Korean and American participants. In addition, Table 5 provides the correlation coefficient among the main variables per country. To test our hypothesis, the first step, similar to that in Study 1, contained the mean-centered emotional suppression of the self, the perceived level of a spouse’s emotional suppression, and regulatory focus. In addition, the country was included as a covariate in Step 1. The second step included the possible two-way interaction terms followed by a three-way interaction term between the variables in the final step.

**Figure 2:**
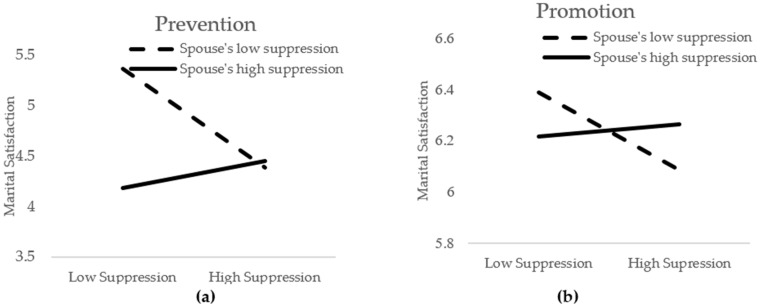
Three-way interaction effects of one’s own level of emotional suppression × perceived level of a spouse’s emotional suppression × regulatory focus on one’s marital satisfaction. (**a**) Two-way interaction effects of one’s own level of emotional suppression × perceived level of a spouse’s emotional suppression for prevention-focused individuals. (**b**) Two-way interaction effects of one’s own level of emotional suppression × perceived level of a spouse’s emotional suppression for promotion-focused individuals.

Table 6 displays the full regression values. Regarding marital satisfaction, the main effects of the three variables and the control variable explained 34.6% (*p* < 0.001) of variance in Step 1, whereas the three sets of two-way interactions contributed an additional 3.2% (*p* < 0.001) of variance in Step 2. Finally, the three-way interaction explained an additional 0.9% (*p* < 0.001) of variance. In Step 3, the results demonstrated the significant main effects of emotional suppression of the self (*b* = −0.08, *p* < 0.001), emotional suppression of the spouse (*b* = −0.09, *p* < 0.001), and regulatory focus (*b* = −0.51, *p* < 0.001). The interaction effects of emotional suppression of the self × emotional suppression of the spouse and regulatory focus × emotional suppression of the spouse were also significant (*b* = 0.08, *p* < 0.001, and *b* = −0.06, *p* < 0.001, respectively). However, these main effects and two-way interactions are qualified by the presence of significant three-way interactions (*b* = 0.03, *t*(960) = 3.71, *p* < 0.001, 95% CI = [0.01, 0.05]), as seen in Figure 2. Additional analyses indicated that this three-way interaction was not qualified by the presence of significant four-way interactions with valence (*b* = 0.02, *t*(614) = 0.80, *p* = 0.42, 95% CI = [−0.03, 0.06]) and gender (*b* = −0.01, *t*(953) = −0.58, *p* = 0.57, 95% CI = [−0.04, 0.02]). In addition, the three-way interaction term remained significant after controlling for marital duration in the regression model (*b* = 0.03, *t*(934) = 3.26, *p* < 0.01, 95% CI = [0.01, 0.04]).

Decomposing the three-way interaction, we first examined prevention-motivated individuals (evaluated at +1 *SD* from the mean). For these individuals, the interaction effect of their own emotional suppression level and the perceived spousal emotional suppression level on marital satisfaction was significant (*b* = 0.13, *t*(960) = 6.96, *p* < 0.001). As in Study 1, the significant interaction term was interpreted by calculating simple slopes at high (+1 *SD*) and low (−1 *SD*) levels of perceived spousal emotional suppression. Specifically, for prevention-focused individuals who viewed their spouses as highly emotionally suppressive, their own emotional suppression level was marginally associated with greater marital satisfaction (*b* = 0.09, *t*(960) = 1.90, *p* = 0.06, 95% CI = [0.00, 0.17]). Conversely, for those who perceived their spouses as less emotionally suppressive, their own emotional suppression level was associated with lower marital satisfaction (*b* = −0.31, *t*(960) = −6.90, *p* < 0.001, 95% CI = [−0.40, −0.22]).

For promotion-motivated individuals (evaluated at −1 *SD* from the mean), the interaction effect of one’s own level of emotional suppression and the perceived level of their spouse’s emotional suppression on marital satisfaction was significant (*b* = 0.04, *t*(960) = 1.93, *p* = 0.05). Again, the significant interaction term was interpreted by calculating simple slopes at high (+1 *SD*) and low (−1 *SD*) levels of perceived spousal emotional suppression. For promotion-focused individuals who perceived their spouses as less emotionally suppressive, being less emotionally suppressive themselves was related to higher marital satisfaction (*b* = −0.10, *t*(960) = −2.12, *p* < 0.05, 95% CI = [−0.19, −0.01]). In contrast, for those who viewed their spouses as highly emotionally suppressive, being less suppressive of emotions was no longer associated with greater marital satisfaction (*b* = 0.01, *t*(960) = 0.30, *p* = 0.76, 95% CI = [−0.08, 0.11]).

In Study 2, two additional analyses were conducted to confirm that the results of the three-way interaction did not differ by country. First, the four-way interaction of one’s emotional suppression × spouse’s emotional suppression × regulatory focus × country was examined, and revealed that the result of the three-way interaction was not qualified by the presence of country (*b* = −0.01, *t*(953) = −0.66, *p* = 0.51, 95% CI = [−0.05, 0.02]). Second, the three-way interaction effect among variables was separately analyzed in Korean and American samples. In both countries, significant three-way interaction effects were obtained: *b* = 0.04, *t*(507) = 2.62, *p* < 0.01, 95% CI = [0.01, 0.07] for Koreans, and *b* = 0.03, *t*(445) = 2.50, *p* < 0.05, 95% CI = [0.01, 0.05] for Americans.

## 7. General Discussion

How does withholding emotional expressions function within marital relationships? According to the present study’s results, the effect depends on one’s self-regulatory processes (prevention vs. promotion) and the extent of the perceived level of spousal emotional suppression. The results of the two studies consistently showed that for prevention-motivated individuals, being suppressive of emotions was positively associated with marital satisfaction but only when their spouses were also perceived as emotionally suppressive. When their spouses were viewed as being emotionally expressive, emotional suppression was negatively associated with marital satisfaction. Conversely, for promotion-motivated individuals, being less emotionally suppressive was positively associated with their marital satisfaction but, again, only when their spouses were also perceived as such. This positive association was mitigated when their spouses were perceived as being suppressive of their emotions. Furthermore, the results in Study 2 revealed that these findings did not vary according to the valence of emotions (i.e., positive and negative).

It is worth noting that the current study found a negative effect of emotional suppression for prevention-motivated individuals when their spouses were perceived as emotionally expressive. Although this negative consequence of incongruent emotional behavior in relationships was unexpected, in-depth investigation is required to understand the effect of the lack of fit in less-ideal situations on marriage. Given that incongruency between the behaviors of spouses leads to marital disruption and distress [38,39], spousal incongruence may be intolerant for those with prevention motivation (compared with those with promotion motivation), who are highly vigilant of threats that signal insecurity and danger in intimate relationships [40,41]. To enhance the understanding of how incongruent patterns of emotional behaviors of spouses function within the framework of regulatory focus, we hope future research could investigate the different mechanisms through which incongruency influences marital outcomes for people with different motivational goals.

Further, the present studies consistently found a significant main effect of regulatory focus on relationship satisfaction, indicating that the overall relationship satisfaction scores were higher for promotion-focused individuals than for prevention-focused ones. In fact, these results are consistent with the widely accepted notion that those who value promotion generally experience more positive outcomes compared with those who focus on prevention [42,43]. However, one must note that this present study did not aim to investigate how prevention-focused individuals might become more or less satisfied in their marital relationships than their promotion-focused counterparts. Instead of comparing the overall well-being of prevention- and promotion-focused individuals, the present study sought to determine which emotional strategies would be more adaptive for these different groups to promote their relationship satisfaction within their own variance.

In this sense, the present study expands the literature by exploring the role of an individual’s motivation in the context of emotional suppression. Although previous research has explained that individuals with different motivations favor different emotional regulation strategies [14], we believe that the present work was the first to empirically demonstrate the interaction between individuals’ emotional regulation strategies and motivations to influence their relationship judgment. Since a person’s motivational orientation guides their daily behaviors, such as their emotional regulation strategies, the fundamental needs underlying the pursuit of different goals within the relationship (stability or advancement [15]) constitute critical factors in predicting relational well-being.

The present study also contributes to understanding the role of congruence within intimate relationships. The results show that the positive effects of one’s emotional regulation strategies on achieving their motivational goals of stability or advancement can manifest mainly when their partners’ emotional behavior is perceived as consistent with those strategies. In line with this assertion, other scholars have highlighted the importance of regulatory fit in dynamic relationships [44]. Since individuals evaluate the quality of their relationships by monitoring how well their relational needs are supported by their partners [29], their perceptions of their partners’ behavior are regarded as an important factor for their perception of the relationship. While many studies have emphasized the importance of a partner’s behaviors, to our knowledge, no research has directly investigated the interactive association between the emotional suppression levels of both partners. Thus, it would be prudent for future studies to examine the interpersonal effects of emotional suppression with consideration for the partner’s emotional suppression.

In addition, the present study suggests implications on the study on emotional suppression and regulatory focus from cross-cultural perspectives. As prevention motivation is more pronounced in Asian cultures than in Western cultures [45], our findings may help with understanding, at a broader level, why emotional suppression is more often found to have positive effects in East Asian cultures [12,46]. Although the present study found that individual differences in motivational orientations account for the differential effects of emotional suppression above and beyond the cultural level, we hope future studies could investigate whether the cultural differences in people’s motivational orientations do in fact explain the different effects of emotional suppression at a cross-cultural level.

Finally, the present study explored the role of emotional valence in the interactive association between emotional suppression and regulatory focus, finding a lack of variation in the effects of the suppression of positive and negative emotions. This implies that emotional suppression is generally an adaptive or maladaptive behavioral strategy depending on one’s regulatory focus with less regard to the valence of emotions. However, an alternative explanation for this null finding of moderating effects is that different types of emotions with varying arousal levels were not investigated separately. Given that emotions have different degrees of arousal [47], emotions with high arousal levels (e.g., distress) and those with low arousal levels (e.g., sadness) may function differently in interpersonal relationships. This necessitates more relevant work to demonstrate the distinctive role of specific types of positive and negative emotions in intimate relationships to obtain a better idea of how regulatory strategies affect emotional suppression.

Although the present study contributes to the findings on the differential effects of emotional suppression in terms of an individual’s motivational orientation, we could not infer any causal relationships between the variables because of certain limitations in this correlational study. Thus, future research efforts may conduct an experimental or longitudinal study exploring whether prevention- and promotion-motivated individuals’ emotional suppression could predict longitudinal changes in marital satisfaction. Further, despite this study’s primary focus on spouses’ perceived level of emotional suppression, it would also be valuable to explore how the fit between two spouses’ actual emotional behavior levels operate under different motivational systems in intimate relationships. Thus, we suggest that future researchers investigate spouses’ actual levels of emotional suppression using dyadic data to expand our results.

## 8. Conclusions

To conclude, the current study sheds light on the inconsistent findings on the adaptiveness of emotional suppression within marriage. Given that individuals have different needs based on their marital dynamics, the present study demonstrates that emotional suppression may not be equally beneficial or detrimental to each individual’s psychological well-being in every case. Considering one’s self-regulatory processes and their spouse’s emotional behavior, the present study lays the foundation for future studies on emotional regulation and regulatory focus.

## Figures and Tables

**Table 1:** Means and standard deviations for Study 1 variables according to age groups.

AGE	Mean (*SD*)			
	21–30 (*N* = 21)	31–40 (*N* = 520)	41–50 (*N* = 454)	More than 51 (*N* = 183)
*N* = 1178 (1 missing) (1) One’s own level of emotional suppression	3.40 (1.40)	3.65 (1.30)	4.00 (1.18)	4.28 (1.12)
(2) Perceived level of a spouse’s emotional suppression	3.74 (1.56)	3.77 (1.26)	3.86 (1.24)	3.94 (1.29)
(3) Regulatory focus	3.71 (1.62)	3.83 (1.58)	4.08 (1.50)	4.48 (1.45)
(4) Marital satisfaction	5.33 (1.62)	5.27 (1.19)	4.86 (1.20)	4.82 (1.21)

**Table 2:** Correlation coefficients among Study 1 variables.

*N* = 1179	(1)	(2)	(3)	(4)
(1) One’s own level of emotional suppression	1	0.35 **	0.47 **	−0.18 **
(2) Perceived level of a spouse’s emotional suppressio		1	0.30 **	0.04
(3) Regulatory focus			1	−0.33 **
(4) Marital satisfaction				1

Correlation (**) is significant at the 0.01 level (2-tailed). No missing values were observed, and all samples were included in the correlational analyses. Appendix A (Table A1) provides the correlation coefficients adjusted for age.

**Table 3:** Hierarchical regression analysis results for Study 1.

		Unstandardized Coefficients		Standardized Coefficients			95% Confidence Interval for B	
		*b*	Std. Error	Beta	*t*	Sig.	Lower Bound	Upper Bound
Step 1	(Constant)	5.04	0.03		152.18	0.00	4.98	5.11
	One’s own Emotional Suppression	−0.08	0.03	−0.08	−2.57	0.01	−0.14	−0.02
	Spousal Emotional Suppression	0.16	0.03	0.17	5.66	0.00	0.11	0.22
	Regulatory Focus	−0.27	0.03	−0.34	−10.97	0.00	−0.32	−0.22
Step 2	(Constant)	4.94	0.04		141.38	0.00	4.87	5.01
	One’s own Emotional Suppression	−0.06	0.03	−0.06	−1.99	0.05	−0.12	−0.00
	Spousal Emotional Suppression	0.14	0.03	0.14	4.98	0.00	0.08	0.19
	Regulatory Focus	−0.26	0.02	−0.33	−10.88	0.00	−0.31	−0.21
	Interaction 1	0.12	0.02	0.20	6.57	0.00	0.09	0.16
	Interaction 2	0.05	0.02	0.09	2.86	0.00	0.02	0.08
	Interaction 3	0.01	0.02	0.01	0.41	0.68	−0.03	0.04
Step 3	(Constant)	4.93	0.04		141.54	0.00	4.86	5.00
	One’s own Emotional Suppression	−0.07	0.03	−0.08	−2.44	0.02	−0.13	−0.01
	Spousal Emotional Suppression	0.10	0.03	0.11	3.49	0.00	0.05	0.16
	Regulatory Focus	−0.27	0.02	−0.34	−11.20	0.00	−0.32	−0.22
	Interaction 1	0.12	0.02	0.19	6.10	0.00	0.08	0.15
	Interaction 2	0.05	0.02	0.09	2.82	0.01	0.02	0.08
	Interaction 3	0.02	0.02	0.03	1.14	0.26	−0.01	0.05
	Interaction 4	0.03	0.01	0.09	2.88	0.00	0.01	0.04

Interaction 1 = one’s own level of emotional suppression × perceived level of a spouse’s emotional suppression; Interaction 2 = one’s own level of emotional suppression × regulatory focus; Interaction 3 = perceived level of a spouse’s emotional suppression × regulatory focus; and Interaction 4 = one’s own level of emotional suppression × perceived level of a spouse’s emotional suppression × regulatory focus. $R^{2}$ = 0.13 for Step 1 (*p* < 0.001); Δ$R^{2}$ = 0.07 for Step 2 (*p* < 0.001); Δ$R^{2}$ = 0.01 for Step 3 (*p* < 0.01).

**Table 4:** Means and standard deviations for Study 2 variables according to age groups.

AGE	Mean (*SD*)			
	21–30 (*N* = 45)	31–40 (*N* = 164)	41–50 (*N* = 205)	More than 51 (*N* = 96)
Korea (*N* = 510, 5 missing) (1) One’s own level of emotional suppression	3.22 (1.60)	3.23 (1.52)	3.63 (1.38)	3.86 (1.39)
(2) Perceived level of a spouse’s emotional suppression	3.61 (1.43)	3.30 (1.45)	3.67 (1.34)	3.67 (1.44)
(3) Regulatory focus	−0.97 (1.29)	−0.83 (1.19)	−0.64 (1.24)	−0.53 (0.94)
(4) Marital satisfaction	5.60 (1.49)	5.49 (1.25)	4.92 (1.47)	4.91 (1.43)
**AGE**	**21–30 (*N* = 71)**	**31–40 (*N* = 169)**	**41–50 (*N* = 116)**	**More than (*N* = 81)**
America (*N* = 437, 16 missing)				
(1) One’s own level of emotional suppression	3.83 (1.70)	2.94 (1.62)	3.35 (1.59)	3.28 (1.72)
(2) Perceived level of a spouse’s emotional suppression	3.86 (1.74)	2.81 (1.53)	2.93 (1.42)	2.68 (1.32)
(3) Regulatory focus	−1.05 (1.56)	−1.79 (1.82)	−1.63 (1.94)	−2.19 (1.55)
(4) Marital satisfaction	5.75 (1.29)	5.74 (1.36)	5.48 (1.54)	6.17 (1.07)

**Table 5:** Correlation coefficients among Study 2 variables.

(*N* = 515 for Korea, *N* = 453 for America)	(1)	(2)	(3)	(4)
(1) One’s own level of emotional suppression	1	0.41 **	0.19 **	−0.22 **
(2) Perceived level of a spouse’s emotional suppression	0.46 **	1	0.10 **	−0.20 **
(3) Regulatory focus	0.37 **	0.28 **	1	−0.57 **
(4) Marital satisfaction	−0.29 **	−0.19 **	−0.57 **	1

Correlation (**) is significant at the 0.01 level (2-tailed). Correlations for Koreans are presented above the diagonal, and the correlations for Americans are presented below. No missing values were observed, and all samples were included in the correlational analyses. Appendix A (Table A2) provides the correlation coefficients adjusted for age.

**Table 6:** Hierarchical regression analysis results for Study 2.

		Unstandardized Coefficients		Standardized Coefficients			95% Confidence Interval for B	
		*b*	Std. Error	Beta	*t*	Sig.	Lower Bound	Upper Bound
Step 1	(Constant)	5.33	0.12		43.83	0.00	5.10	5.57
	Country	0.07	0.08	0.03	0.91	0.36	−0.08	0.23
	One’s own Emotional Suppression	−0.07	0.03	−0.07	−2.41	0.02	−0.12	−0.01
	Spousal Emotional Suppression	−0.05	0.03	−0.05	−1.84	0.07	−0.11	0.00
	Regulatory Focus	−0.49	0.03	−0.54	−18.57	0.00	−0.54	−0.44
Step 2	(Constant)	5.26	0.12		44.06	0.00	5.02	5.49
	Country	0.10	0.08	0.08	1.30	0.20	−0.05	0.25
	One’s own Emotional Suppression	−0.08	0.03	−0.08	−2.88	0.00	−0.13	−0.02
	Spousal Emotional Suppression	−0.07	0.03	−0.07	−2.41	0.02	−0.12	−0.01
	Regulatory Focus	−0.48	0.03	−0.53	−18.51	0.00	−0.53	−0.43
	Interaction 1	0.08	0.01	0.16	5.90	0.00	0.06	0.11
	Interaction 2	−0.03	0.02	−0.06	−1.98	0.05	−0.06	0.00
	Interaction 3	−0.06	0.02	−0.10	−3.51	0.00	−0.09	−0.03
Step 3	(Constant)	5.29	0.12		44.50	0.00	5.06	5.53
	Country	0.09	0.08	0.03	1.12	0.26	−0.07	0.24
	One’s own Emotional Suppression	−0.08	0.03	−0.09	−2.99	0.00	−0.13	−0.03
	Spousal Emotional Suppression	−0.09	0.03	−0.09	−3.14	0.00	−0.14	−0.03
	Regulatory Focus	−0.51	0.03	−0.57	−18.79	0.00	−0.57	−0.46
	Interaction 1	0.08	0.01	0.16	5.91	0.00	0.06	0.11
	Interaction 2	−0.03	0.02	−0.05	−1.56	0.12	−0.06	0.01
	Interaction 3	−0.06	0.02	−0.10	−3.53	0.00	−0.09	−0.03
	Interaction 4	0.03	0.01	0.11	3.71	0.00	0.01	0.05

Interaction 1 = one’s own level of emotional suppression × perceived level of a spouse’s emotional suppression; Interaction 2 = one’s own level of emotional suppression × regulatory focus; Interaction 3 = perceived level of a spouse’s emotional suppression × regulatory focus; and Interaction 4 = one’s own level of emotional suppression × perceived level of a spouse’s emotional suppression × regulatory focus. $R^{2}$ = 0.346 for Step 1 (*p* < 0.001); Δ$R^{2}\;$ = 0.032 for Step 2 (*p* < 0.001); and Δ$R^{2}$ = 0.009 for Step 3 (*p* < 0.001).

## Data Availability

The data that support the findings of this study are available on request from the corresponding author. The data are not publicly available due to privacy or ethical restrictions.

## Appendix A

**Table A1:** Correlation coefficients adjusted for age in Study 1.

	(1)	(2)	(3)	(4)
(1) One’s own level of emotional suppression	1	0.35 **	0.45 **	−0.15 **
(2) Perceived level of a spouse’s emotional suppression		1	0.30 **	0.05
(3) Regulatory focus			1	−0.31 **
(4) Marital satisfaction				1

Correlation (**) is significant at the 0.01 level (2-tailed).

**Table A2:** Correlation coefficients adjusted for age in Study 2.

	(1)	(2)	(3)	(4)
(1) One’s own level of emotional suppression	1	0.40 **	0.17 **	−0.19 **
(2) Perceived level of a spouse’s emotional suppression	0.44 **	1	0.09	−0.18 **
(3) Regulatory focus	0.37 **	0.25 **	1	−0.56 **
(4) Marital satisfaction	−0.31 **	−0.18 **	−0.57 **	1

Correlation (**) is significant at the 0.01 level (2-tailed). Correlations for Koreans are presented above the diagonal, and the correlations for Americans are presented below.
